# MIREyA: a computational approach to detect miRNA-directed gene activation

**DOI:** 10.12688/f1000research.28142.2

**Published:** 2021-08-26

**Authors:** Anna Elizarova, Mumin Ozturk, Reto Guler, Yulia A. Medvedeva

**Affiliations:** 1 Group of Regulatory Transcriptomics and Epigenomics, Research Center of Biotechnology, Institute of Bioengineering, Russian Academy of Sciences, Moscow, 117312, Russian Federation; 2 Department of Biological and Medical Physics, Moscow Institute of Physics and Technology (National Research University), Dolgoprudny, 141701, Russian Federation; 3 International Centre for Genetic Engineering and Biotechnology, Cape Town, Cape Town, 7925, South Africa; 4 Department of Pathology, University of Cape Town, Institute of Infectious Diseases and Molecular Medicine (IDM), Division of Immunology and South African Medical Research Council (SAMRC) Immunology of Infectious Diseases, Faculty of Health Sciences, Cape Town, 7925, South Africa; 5 Wellcome Centre for Infectious Diseases Research in Africa (CIDRI-Africa), Institute of Infectious Disease and Molecular Medicine (IDM), Faculty of Health Sciences, University of Cape Town, Cape Town, 7925, South Africa

**Keywords:** microRNA, miRNA, non-coding RNA, enhancer, regulator, macrophage, tuberculosis

## Abstract

Emerging studies demonstrate the ability of microRNAs (miRNAs) to activate genes via different mechanisms. Specifically, miRNAs may trigger an enhancer promoting chromatin remodelling in the enhancer region, thus activating the enhancer and its target genes. Here we present MIREyA, a pipeline developed to predict such miRNA-gene-enhancer trios based on an expression dataset which obviates the need to write custom scripts. We applied our pipeline to primary murine macrophages infected by *Mycobacterium tuberculosis *(HN878 strain) and detected Mir22, Mir221, Mir222, Mir155 and Mir1956, which could up-regulate genes related to immune responses. We believe that MIREyA is a useful tool for detecting putative miRNA-directed gene activation cases. MIREyA is available from: https://github.com/veania/MIREyA

## Introduction

Conventionally, microRNAs (miRNAs) are considered to suppress gene expression through RNA interference (RNAi) by binding complementarily to mRNAs, forming a RISC complex, and causing RNA degradation. ^1^ However, recent studies provide evidence that some miRNAs act in the opposite way – stimulating gene activation. Numerous studies have demonstrated the ability of miRNAs to up-regulate genes by targeting their promoters. ^2, 3^ Ago1 from miRNA-Ago complex associates with the Ccnb1 promoter and miR-744 induces enrichment of RNA Pol II and H3K4me3 at the Ccnb1 transcription start site. ^3^ MiRNA let-7i interacts with the TATA-box of IL-2 gene and stimulates transcription initiation by contributing to the assembly of the pre-initiation complex. ^4^ Relatively fewer miRNAs demonstrated the ability to unconventionally target and activate enhancers, thus inducing genes regulated by these enhancers. MiR-24-1 acts as a modulator of the chromatin state of an enhancer. Furthermore, it increases p300 and RNA Pol II binding at the enhancer locus. The miR-24-1 actually originates from the enhancer locus. However, some genes regulated by other enhancers are also expressed at higher levels when miR-24-1 is transfected, and the enhancers of induced genes contain a sequence similar to the seed of the miRNA. ^5^ These observations suggest that other miRNAs might trigger enhancers and activate gene expression.

When miRNAs function as activators in a nucleus, different targeting mechanisms are possible: miRNA:DNA Watson-Crick duplex formation as well as miRNA:DNA Hoogsteen triple helix formation. Nuclear miRNA target prediction tools utilize are based on the idea that miRNA:DNA interaction requires an intact seed region. ^6^ MicroPIR2 predicts targets in mouse and human promoter regions. ^7^ Trident predicts miRNA:DNA Hoogsteen-type base pairings. ^8^ Some tools designed to predict conventional miRNA targets may also be utilized to find nuclear activational targets, e.g. miRanda. ^9^

In this work, we report MIREyA (MIRnas functioning through Enhancer Activation), a pipeline for detection of miRNAs and their gene targets up-regulated through triggering their enhancer in the provided expression dataset. We applied MIREyA in order to identify and characterize activational miRNAs in *Mycobacterium tuberculosis* (Mtb) infected macrophage dataset. MiRNAs are important regulators in macrophage responses during Mtb infection that may act as host immunity agents as well as a tool exploited by pathogen agents to manipulate host cell pathways. ^10^ Yet, only the classical role of miRNA has been investigated so far in the context of Mtb infection. ^11– 19^ Although multiple studies have shown the possibility of the activational role of miRNAs, this potential remains neglected in the study of miRNAs in bacterial infections. MIREyA found several miRNAs, which have not been shown to be functional in TB yet, suggesting it could be useful to find candidate activational miRNAs for further experimental validation. Mir155 has previously been shown to act as a negative regulator of essential mRNAs during TB, ^20^^,^^21^ but not as an activator.

## Methods

### Main steps of the algorithm

MIREyA aims to detect miRNAs with the potential to upregulate a gene via activation of its enhancer. It consists of three major steps:

1) The algorithm detects miRNA bound to the enhancers. This step is implemented with three different approaches described below.
2) The algorithm selects genes regulated by the enhancers selected in step 1). For this step the output from the first step is required as well as a table with enhancer:gene pairs where an enhancer is assumed to regulate the corresponding gene.
3) The algorithm calculates the Spearman’s correlation coefficient (SCC) between the expression levels of miRNAs and genes regulated by corresponding enhancers selected in step 1), and estimates the p-value of the SCC with a Benjamini-Hochberg correction using the number of the miRNA:gene pairs for one miRNA (FDR < 0.05). The input data is gene expression data with sample size ≥ 8 and the output of the previous step.

**Figure 1.**
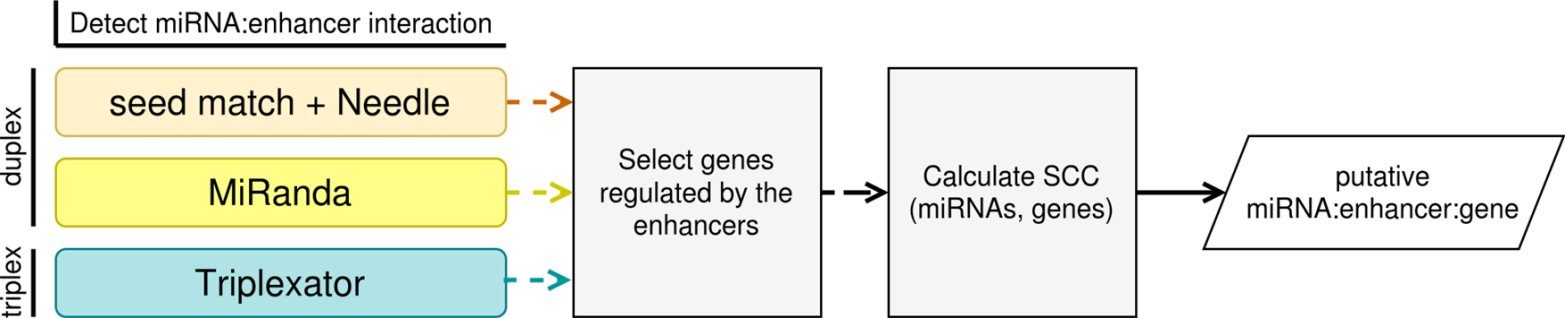
The outline of the implemented algorithm.

Figure 1 illustrates the workflow of our algorithm implemented in Python and R.

### Prediction of miRNA-enhancer interaction

We speculate that in order to activate an enhancer, miRNA should bind to enhancer DNA. Since the mechanism of such binding is unclear, we decided to implement several reasonable prediction strategies. The first two strategies assume that miRNA binds to DNA forming an RNA:DNA double helix, while the third assumes RNA:DNA triple helix formation.

1) The first approach is to select an enhancer containing an exact match of the user-provided seed sequence of a miRNA, then expand each seed by 14 bp of the corresponding mature miRNA and align it to the enhancer with Needle tool ^22^ and keep only enhancers with the percent identity (PI) > 50 (PI defined as a percent of matches between miRNA and DNA region).
2) The second approach is to scan miRNA sequences against enhancer sequences and detect potential target sites with MiRanda. ^9^
3) The third approach is to predict RNA:DNA triplexes between miRNAs and enhancers with Triplexator tool. ^23^ We relaxed the error-rate and lower-length-bound Triplexator default parameters in order to adjust the algorithm to work with extremely short miRNA sequences (error-rate=19, lower-length-bound=11).

The approaches are interchangeable, also the user can merge the results of all approaches to reflect multiple mechanisms of potential miRNA:DNA binding.

The main script to run the pipeline is src/run_mireya.py. Input and output files depend on choice of one of previously described approaches to predict miRNA-enhancer interaction and must be specified with the following flags:

-d/ --detection_mir_enh_interaction: approach to predict miRNA-enhancer interaction, accepts one of three possible values: seed_match_needle / miranda / triplexator

-e/ --enhancers: path to a fasta file with sequences of enhancers of interest

-o/ --output: full path to output directory

-ge/ --gene_expression: path to .tsv file with normalized gene expression

-me/ --mirnas_expression: path to .tsv file with expression of miRNAs of interest

-ei/ --enh_gene_interaction: path to .tsv file with enhancers and corresponding genes they are assumed to regulate

-m/ --mature_mirnas: path to a fasta file with sequences of mature miRNAs of interest

Pipeline mode with the first approach to predict miRNA-enhancer interaction (-d seed_match_needle) requires additional input data which must be provided with flags:

-g/ --genome: path to a folder with fasta files with complete genome of the organism of interest: one chromosome per file

-s/ --seeds_mirnas_forward: path to a tab-delimited file with sequences of seeds of miRNAs of interest, without header with 2 columns: 1) general name of mirnas; 2) their seeds as DNA sequences (make sure that U are replaced with T)

-sr/ --seeds_mirnas_reverse_compl: path to a tab-delimited file with reverse complementary sequences of seeds of miRNAs of interest, without header with 2 columns: 1) general name of mirnas; 2) their seeds as DNA sequences (make sure that U are replaced with T)

-eb/ --enhancers_bed: path to bed file with coordinates of enhancers of interest

-ms/ --mature_mirnas_separate: path to a directory containing directories with mature miRNA sequences: one folder per miRNA containing fasta files with one mature sequence per file. Each directory with mature mirna fastas must be named exactly as in other input files. Names of fasta files will be used further as "mature_mirna" column in result tables.

MiRNAs must have the same names in -ge, -me, -ei, -s, -sr input data and for folders in -ms argument. Please, use example files to make sure the input is in correct format. MiRNA names for mature sequences must be the same in -m and -ms arguments.

Examples of command to run the main script, one for each approach to predict miRNA-enhancer interaction (names of files coinside with names of example input files in repository):

1. python src/run_mireya.py -d seed_match_needle -e data/enhancers.macrophages.Mtb.mm9.fasta -o out/seed_match_needle_out/ -ge data/DE_gene_expression.tsv -me data/DE_mirnas_expression.tsv -ei data/enh.gene.assoc.sign.tsv -m data/DE_mirna_mature_seqs.fa -g data/db -s data/seeds_seq_forward_short -sr data/seeds_seq_reverse_compl_short -eb data/enhancers.macrophages.Mtb.bed -ms data/mature_seqs/
2. python src/run_mireya.py -d miranda -e data/enhancers.macrophages.Mtb.mm9.fasta -o out/miranda_out/ -ge data/DE_gene_expression.tsv -me data/DE_mirnas_expression.tsv -ei data/enh.gene.assoc.sign.tsv -m data/DE_mirna_mature_seqs.fa
3. python src/run_mireya.py -d triplexator -e data/enhancers.macrophages.Mtb.mm9.fasta -o out/triplexator_out -ge data/DE_gene_expression.tsv -me data/DE_mirnas_expression.tsv -ei data/enh.gene.assoc.sign.tsv -m data/DE_mirna_mature_seqs.fa

Output file is called mir_enh_gene_trios.tsv. Example output file is placed in the out/directory of repository. One line in the output file corresponds to one trio of a mature miRNA, an enhancer and a gene (“mature_mirna”, “Gene.Name”, “enhancer” columns). Columns “corr (miRNA, gene)”and “p.value adj” correspond to Spearman’s correlation coefficient of the miRNA and the gene expression and FDR. “PI” column corresponds to percent identity and is present only in the output of seed_match_needle approach.

### Implementation

The pipeline is implemented as Python, R and bash scripts, and can be run with a master script run_mireya.py.

### Operation

Python>=3.5 and r-base are expected to be pre-installed. Besides, two modes of the pipeline require the following tools installed: MiRanda, Triplexator. The pipeline was tested in Ubuntu and Ubuntu-based linux systems (Ubuntu>=16.04).

## Use case

We applied MIREyA to three time-series (0, 4, 12, 24, 48, 96 hours) expression datasets (CAGE) of mouse bone marrow-derived macrophages infected with hypervirulent Beijing/W lineage *Mycobacterium tuberculosis* (Mtb) HN878 strain, 2-4 replicates per time point. ^24^ Each dataset corresponds to the time series after infection for macrophages of different phenotypes: not pre-stimulated (M0), interferon-γ stimulated (M1-polarized) and interleukin-4/interleukin-13 stimulated (M2-polarized). Only differentially expressed (DE) miRNAs and genes were considered. We obtained enhancer-gene interactome from ^25^ where an enhancer is predicted to regulate a gene if their expression levels correlate significantly and they belong to the same topologically associated domain (TAD).

We searched for candidate enhancers targeted by miRNAs with all three methods described previously and merged the results for further steps. CAGE enables one to estimate expression at the promoter level, while enhancers are associated with whole genes. As a proxy of gene expression, we used either an expression value of a promoter with the highest SCC (a) or summed up the expression values of all promoters of the gene (b). To reduce the number of false positive predictions, we selected among miRNA:enhancer duplexes only such cases where (1) a duplex was predicted both by miRanda and Needle-based approach; (2) an enhancer was associated with several genes since in the original paper on miRNA-activated enhancers ^5^ one nuclear miRNA affected expression of multiple genes regulated by the triggered enhancer; (3) the miRNA-gene pair was obtained in both ways (a and b) to estimate expression of the gene. We also added miRNA-gene pairs with highly correlated expression levels (SCC ≥ 0.8) in any of four combinations of two methods to detect miRNA:enhancer interactions (seed match + Needle and miRanda) and two approaches to treat expression of different promoters (a and b). Among predicted miRNA:enhancer triplexes we selected cases where (1) an enhancer was associated with multiple genes; (2) both approaches to estimate gene expression (a and b) yielded this triplex.

## Results

**Figure 2.**
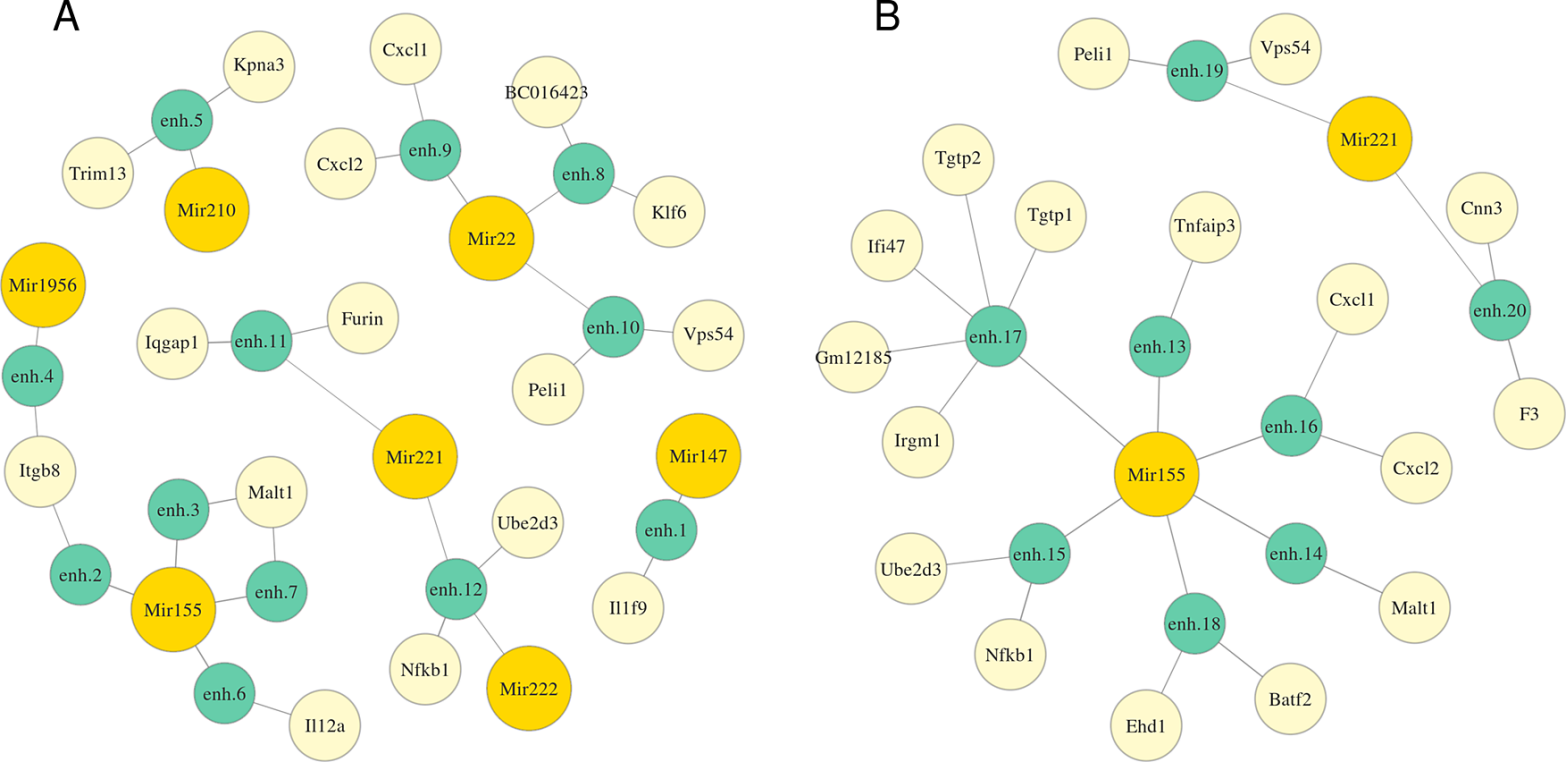
Predicted regulation graphs of miRNAs via RNA:DNA duplex (A) or RNA:DNA triplex formation (B) with an enhancer for M0 macrophages. The length of an edge between a miRNA and an enhancer or between an enhancer and a gene is in inverse proportion to the average correlation between the expression of the miRNA and the target gene or the enhancer and the gene respectively. Full coordinates of enhancers are available in Table S1.

We applied MIREyA to three time-series datasets of Mtb-infected macrophages with 3 different phenotypes prior to infection. In M0 macrophages 10 miRNAs were differentially expressed (DE) in at least one time point compared to the state before infection (0h), in M1 there was no DE miRNA, while in M2 only Mir1956 was DE. Figure 2 and Table S1 ( *Extended data* ^43^) represent detected miRNA-enhancer-gene trios for M0 macrophages ( *Extended data:* Table S2 for M2 macrophages ^43^). We investigated the functions of the obtained genes and miRNAs and confirmed that many of them might be involved in the response to Mtb infection. We detected Mir155, which is vastly studied, and known to subvert autophagy in human dendritic cells ^20^ and to be a potential diagnostic marker of active tuberculosis. ^21^ Other miRNAs and their targets which we consider promising for further investigation are summed in Tables 1 and 2. Mir22, Mir221, Mir222 are annotated with high confidence. ^26^ Expression of only some promoters of Klf6 and BC016423 genes correlates with Mir22 expression, but we included them, because they have predicted triplexes with three different enhancers.

**Table 1.** The most promising miRNAs predicted for M0 infected BMDMs to upregulate genes via enhancer activation.

miRNAs	genes	number of enhancers	mechanism of interaction of enhancer:miRNA
Mir22	Klf6, BC016423	3	duplex, triplex
Mir221, Mir222, Mir155	Nfkb1, Ube2d3	2	duplex, triplex
Mir22, Mir221	Peli1	2	duplex, triplex
Mir22, Mir221	Cxcl1, Cxcl2	2	duplex, triplex
Mir155	Malt1	2	duplex

**Table 2.** The most promising miRNAs predicted for M2 pre-stimulated infected BMDMs to upregulate genes via enhancer activation.

miRNAs	genes	number of enhancers	mechanism of interaction of enhancer:miRNA
Mir1956	Ccrl2	1	duplex
Mir1956	Dot1l	5	duplex
Mir1956	Cd14	1	duplex
Mir1956	Rab20	1	duplex
Mir1956	Ticam1	1	duplex
Mir1956	Tnfaip3 (A20)	2	duplex

We reconstructed regulatory networks for these miRNAs based on miRNA-enhancer-gene trios and investigated their potential role in the response to Mtb infection. Mir22-activated gene network is highly likely to be involved in Mtb response. Klf6, a potential target of Mir22, is a transcription factor essential for macrophage motility ^27^ and plays an important role in the regulation of macrophage polarization promoting M1 phenotype cooperatively with NF-κB. ^28^ Nfkb1 gene, a putative target of miRNAs Mir221, Mir222 and Mir155, encodes a subunit of NF-κB protein complex, a master transcription factor in macrophage immune responses. The human ortholog of Ube2d3, potentially regulated by the same miRNAs, facilitates polyubiquitination of NFKBIA (a member of the NF-kappa-B inhibitor family) stimulating its subsequent degradation. ^29^ A detected target of both Mir22 and Mir221, Peli1 regulates the NF-κB activity negatively and attenuates the induction of proinflammatory cytokines in T-cells. ^30^ Cxcl1 and Cxcl2, detected targets of both Mir22 and Mir221, are chemoattractants for neutrophils contributing to tissue inflammation. ^31^ Malt1, potentially up-regulated by Mir155, is known to activate NF-κB in lymphocytes. ^32^ Among targets of Mir1956 detected in M2 macrophages dataset we found the Ccrl2 gene encoding a chemokine receptor-like protein which is expressed at high levels in primary neutrophils and primary monocytes. Another Mir1956 target, Dotl1, is an H3K79 methyltransferase which facilitates the expression of IL-6 and IFN-β in macrophages. ^33^ Cd14 leads to NF-κB activation and inflammatory response, ^34^ Cd14 KO mice infected with Mtb are protected due to reduced inflammatory responses at the chronic stage. ^35^ Rab20 plays a role in the maturation and acidification of phagosomes and the fusion of phagosomes with lysosomes during mycobacterial infection. ^36^ Ticam1 is involved in native immunity against pathogens: it interacts specifically with toll-like receptor 3, activates NF-κB. ^37^ Tnfaip3 (A20) is an important regulatory protein that down-regulates NF-κB activity. ^38^

**Figure 3.**
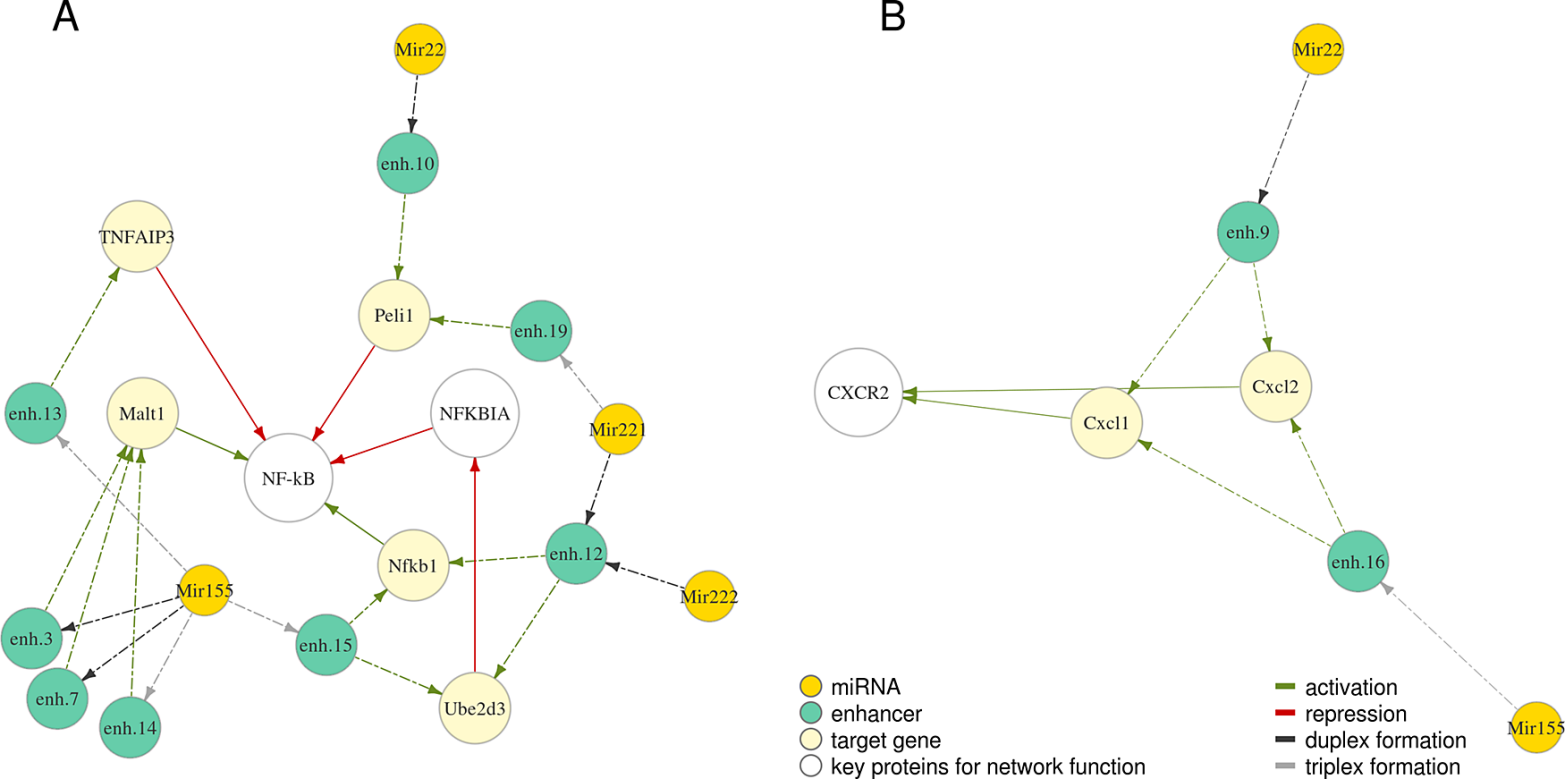
MiRNA-enhancer-gene trios detected for M0 macrophages include genes with related functions and form subnetworks A (related to NF-kB) and B (both genes encode chemokines which signal through CXCR2). Relationships are depicted with arrows of a different colour: green – activation, red – repression. Dashed arrows indicate predicted relationships, solid – known from published studies and described previously. Full coordinates of enhancers are available in Table S1.

We further investigated protein networks associated with the selected miRNAs. Proteins regulated by miRNAs Mir22, Mir155, Mir221, Mir222 are involved in regulation of NF-κB – a vital orchestrator of the response of the innate immune cells to pathogens ^39^ ( Figure 3A). Cxcl1 and Cxcl2 regulated by Mir22 and Mir155 ( Figure 3B) are chemokines which signal through CXC receptor 2 to attract neutrophils to the place of inflammation, which is essential to control tissue infection. ^31^

## Discussion

MIREyA aims to easily find candidate activating miRNAs which trigger an enhancer and genes up-regulated by the enhancer. New emerging methods for studying RNA–DNA interactome detect previously unknown miRNAs bound to chromatin ^40, 41^ which might be promising for further experimental investigation in terms of understanding complex gene regulation networks in detail. Although high-throughput data on RNA:DNA interactions in several cell types but not macrophages are available now, we could not confirm MiRNAs detected with MIREyA for Mtb infection using either RADICL-seq ^41^ or GRID-seq, ^42^ which is unsurprising since the ncRNA:DNA interactions are highly cell-type-specific. ^41^

Although the implemented algorithm considers multiple aspects of the suggested mechanism of gene activation, several factors remain unaccounted for. To run MIREyA a user requires *a priori* knowledge of enhancer-gene interactions. A common fast computational approach to determine gene-enhancer pairs is to use genes and enhancers co-localized in the genome, ignoring the long-distance spatial interactions. One of solutions is the approach suggested in: ^25^ to calculate the correlation between expression of genes and enhancers which belong to the same TAD.

Although the exact mechanism of RNA activation of enhancers remains unclear, we do know that miRNA as a mediator facilitates epigenetic modifications in the enhancer region. At this stage, MIREyA does not consider chromatin availability or any other epigenetic information. In order to reduce the number of false positive predictions, nuclear localisation of specific predicted miRNAs should be validated experimentally.

Despite the discussed limitations, using MIREyA we detected several promising miRNA candidates. We suggest that MIREyA provides a promising approach to select miRNAs which up-regulate genes by triggering enhancers for further experimental validation.

## Conclusion

Our method extends the study of activational miRNAs and provides a basis for further research. The use case on Mtb-infected macrophages demonstrates the possibility of existence of novel miRNAs up-regulating gene expression.

## Data availability

### Underlying data

The CAGE time series expression datasets from the use case are available at https://fantom.gsc.riken.jp/data/ in mm9 Phase 2 release and can be selected to download with FANTOM5 Table Extraction Tool by «macrophage, TB infection» key words.

### Extended data

Zenodo: MIREyA: Extended data, http://doi.org/10.5281/zenodo.4549445. ^43^

This project contains the following extended data:

- Table S1: Detected miRNA-enhancer-gene trios for M0 macrophages
- Table S2: Detected miRNA-enhancer-gene trios for M2 macrophages

Data are available under the terms of the Creative Commons Zero “No rights reserved” data waiver (CC0 1.0 Public domain dedication).

## Software availability

Pipeline available from: https://github.com/veania/MIREyA.

Archived pipeline as at time of publication: https://doi.org/10.5281/zenodo.5082988. ^44^

License: Open Software License 3.0
