# Supplementary material for: MIREyA: a computational approach to detect miRNA-directed gene activation
Source: F1000Res. 2021 Aug 26;10:249. Originally published 2021 Mar 29. [Version 2] doi: 10.12688/f1000research.28142.2 (PMC8411277; doi:10.12688/f1000research.28142.2)
Supplement: Peer review history

# Peer review: Reviewer response for version 2

**DOI:** 10.5256/f1000research.59225.r92860

**Published:** 2021-09-01

**Authors:** Chaogang Shao

**Affiliations:** 1 College of Life Sciences, Huzhou University, Huzhou, China

My questions have been answered.

Are the conclusions about the tool and its performance adequately supported by the findings presented in the article?

Yes

Is the rationale for developing the new software tool clearly explained?

Yes

Is the description of the software tool technically sound?

Yes

Are sufficient details of the code, methods and analysis (if applicable) provided to allow replication of the software development and its use by others?

Yes

Is sufficient information provided to allow interpretation of the expected output datasets and any results generated using the tool?

Yes

Reviewer Expertise:

miRNA data mining, bioinformatics

I confirm that I have read this submission and believe that I have an appropriate level of expertise to confirm that it is of an acceptable scientific standard.

# Peer review: Reviewer response for version 1

**DOI:** 10.5256/f1000research.31129.r87527

**Published:** 2021-07-05

**Authors:** Chaogang Shao

**Affiliations:** 1 College of Life Sciences, Huzhou University, Huzhou, China

The authors have developed a pipeline, MIREyA, to predict miRNA gene-enhancer trios based on an expression dataset. This manuscript covers an important aspect of miRNA-target research by presenting a useful tool that technically sound. I have some questions:

1. A newly developed software needs to prove its function is correct before it is applied. However, the manuscript lacks this important part. Though the authors applied the pipeline to primary murine macrophages infected by Mycobacterium tuberculosis and detected some miRNAs could up-regulate genes related to immune responses, these predicted results need to be proved by experiments. At present, we can’t determine whether these regulatory relationships are correct, so the reliability of MIREyA can not be proved. The authors can use the published miRNAs with activation function, such as MiR-24-1, miR-744 and MiRNA let-7i, to verify the reliability of the pipeline.
2. Are there any miRNAs coming from primary murine macrophases affected by Mycobacterium tuberculosis that can bind to enhancers but reduce gene expression?
3. “keep only enhancers with the percent identity (PI) > 0.5 (PI defined as a percent of matches between miRNA and DNA region)”. Will 0.5 be too low to increase false positives? What is the basis for the author to choose the parameter?
4. Are there any other sRNAs that share very similar sequence to miRNA and express even higher than miRNA in cells? These sRNAs may be involved in the regulation of enhancers too. Whether authors can improve the software to find these co-regulated sRNAs from high throughput sequence data, so as to build a more complete regulatory network.

Are the conclusions about the tool and its performance adequately supported by the findings presented in the article?

Yes

Is the rationale for developing the new software tool clearly explained?

Yes

Is the description of the software tool technically sound?

Yes

Are sufficient details of the code, methods and analysis (if applicable) provided to allow replication of the software development and its use by others?

Yes

Is sufficient information provided to allow interpretation of the expected output datasets and any results generated using the tool?

Yes

Reviewer Expertise:

miRNA data mining, bioinformatics

I confirm that I have read this submission and believe that I have an appropriate level of expertise to confirm that it is of an acceptable scientific standard, however I have significant reservations, as outlined above.

# Response

**Published:** 2021-08-02

**Authors:** Anna Elizarova

**Affiliations:** Research Center of Biotechnology, Institute of Bioengineering, Russian Academy of Sciences, Russian Federation

Thank you for leaving the review.

 As for the questions:

- A newly developed software needs to prove its function is correct before it is applied. However, the manuscript lacks this important part. Though the authors applied the pipeline to primary murine macrophages infected by Mycobacterium tuberculosis and detected some miRNAs could up-regulate genes related to immune responses, these predicted results need to be proved by experiments. At present, we can’t determine whether these regulatory relationships are correct, so the reliability of MIREyA can not be proved. The authors can use the published miRNAs with activation function, such as MiR-24-1, miR-744 and MiRNA let-7i, to verify the reliability of the pipeline.

To find already studied miRNAs with this tool, we need an expression dataset for the same cell types and conditions as in the references. MiRNA expression is highly dependent on cell type and conditions, thus any other expression dataset would have a different set of expressed miRNAs which might not include miRNAs studied in the papers. Another limitation is that the expression dataset must include at least 8 samples so that correlation analysis would make sense. For now we are not aware of such a complete dataset. 

 In case of MiR-24-1, there was no expression dataset to run our pipeline, but the first step of the algorithm (miRNA/enhancer interaction prediction) would automatically find the enhancer from the article: the miRNA sequence is apparently present in the sequence of the enhancer in the reference 3 as the enhancer contains a gene sequence of the miRNA. We found an article ( doi.org/10.1038/srep12987) reporting gene activation by miR-744 and an article (doi.org/10.1073/pnas.1803384115) reporting similar activation by miR-744 but the authors did not demonstrate that these miRNAs do that through enhancer activation. Lack of a known enhancer in a regulatory circuit makes the use of MIREyA impossible. 

- Are there any miRNAs coming from primary murine macrophases affected by Mycobacterium tuberculosis that can bind to enhancers but reduce gene expression?

Since we focused on the activatory role of miRNA, we believe that the analysis of repressor miRNAs bound to enhancers is beyond the scope of the current paper.

- “keep only enhancers with the percent identity (PI) > 0.5 (PI defined as a percent of matches between miRNA and DNA region)”. Will 0.5 be too low to increase false positives? What is the basis for the author to choose the parameter?

We believe that 0.5 is high enough. We chose the threshold by comparison with experimentally validated examples of miRNA interaction with RNA, where the minimum 11/22 miRNA bases represented an exact match.  We hypothesized that such PI would be sufficient for miRNA interaction with DNA.

- Are there any other sRNAs that share very similar sequence to miRNA and express even higher than miRNA in cells? These sRNAs may be involved in the regulation of enhancers too. Whether authors can improve the software to find these co-regulated sRNAs from high throughput sequence data, so as to build a more complete regulatory network.

So far we have not seen any article describing sRNAs with similar to miRNA properties which are able to regulate enhancers. We believe that for now this suggestion is beyond the scope of this work.

# Peer review: Reviewer response for version 1

**DOI:** 10.5256/f1000research.31129.r87525

**Published:** 2021-06-29

**Authors:** Xiangfu Zhong

**Affiliations:** 1 Karolinska Institutet, Stockholm, Sweden

**Summary:**

 The manuscript describes a python pipeline to predict the interaction between miRNA, gene and enhancer, based on the expression data. Their interaction may help us have a better understanding of miRNA regulation mechanisms.

**Comments and suggests for improvement:**

1. Page 3, the methods section, the third step, " *the input data is gene expression data with sample size *>=8". Does this sample size requirement include replicates? Why does MIREyA require this number of sample size?
2. Page 5, in the ”Use case” section, " *2-3 replicates per time point"*. However, the reference 24 says " *Three-four biological replicates were prepared per time point".*
3. Can the authors provide some script or functions for users to process the raw data into MIREyA input format? For example, the first column in *data/DE_mirnas_expression.tsv*, is the miRNA names, but not in the format of mostly used.
4. The example expressions are normalized, it would be clear to mention when explaining *-ge* on page 4.
5. Improve the logging and exit message of running the pipeline. 

**Optional:**

1. The current code looks like in the script format, wrapping them into package and release to pypi may improve the package popularity and availability. The code looks much like customer scripts for analysis for case use data, generation of them into wider usage. 
2. Some genes have multiple copies across the genome, when calculating the correlation between gene expression and enhancer/miRNA, will copy/genomic coordinate be given to the correlation test? At least 3 line expression data about Ccdc59 in the example expression data data/DE_gene_expression.tsv, which one is used in the correlation test?
3. I could not reproduce the first example 1 on page 5:

- git clone git@github.com:veania/MIREyA.git
- Rename the out folder within this repo code, then create another out for testing,
- python src/run_mireya.py -d seed_match_needle -e data/enhancers.macrophages.Mtb.mm9.fasta -o out/seed_match_needle_out/ -ge data/DE_gene_expression.tsv -me data/DE_mirnas_expression.tsv -ei data/enh.gene.assoc.sign.tsv -m data/DE_mirna_mature_seqs.fa -g data/db -s data/seeds_seq_forward_short -sr data/seeds_seq_reverse_compl_short -eb data/enhancers.macrophages.Mtb.bed -ms data/mature_seqs/

  

 First error in step 1:

- Error: Unable to open file out/seed_match_needle_out//Mir146_seed_pos_in_genome. Exiting.

Even with this error, the pipeline keeps running without exit.  For the step 2, I got another error:

- File 'Mir146_enh_with_seeds.bed' has size 0. Returning a NULL data.table.
- Execution halted
- Calculation is finished. Please find the results in mir_enh_gene_trios.tsv

Are the conclusions about the tool and its performance adequately supported by the findings presented in the article?

Partly

Is the rationale for developing the new software tool clearly explained?

Yes

Is the description of the software tool technically sound?

Yes

Are sufficient details of the code, methods and analysis (if applicable) provided to allow replication of the software development and its use by others?

Partly

Is sufficient information provided to allow interpretation of the expected output datasets and any results generated using the tool?

Partly

Reviewer Expertise:

bioinformatics, microRNA annotation and target predication, epigenetics

I confirm that I have read this submission and believe that I have an appropriate level of expertise to confirm that it is of an acceptable scientific standard, however I have significant reservations, as outlined above.

# Response

**Published:** 2021-08-02

**Authors:** Anna Elizarova

**Affiliations:** Research Center of Biotechnology, Institute of Bioengineering, Russian Academy of Sciences, Russian Federation

Thank you for leaving the review.

 As for the questions:

1. Page 3, the methods section, the third step, " *the input data is gene expression data with sample size *>=8". Does this sample size requirement include replicates? Why does MIREyA require this number of sample size?

Yes, the stated sample size includes replicates. We added this constraint because the pipeline relies on calculation of correlation coefficient. It is possible to try it on an expression dataset of smaller size but the result might be less reliable.

1. Page 5, in the ”Use case” section, " *2-3 replicates per time point"*. However, the reference 24 says " *Three-four biological replicates were prepared per time point".*

Authors of the original paper prepared up to 4 replicates, but sequenced only 2-4. To check the exact number of replicates sequenced for each time point one can browse FANTOM5 data (https://fantom.gsc.riken.jp/5/) or have a look at Supplementary table 1 in the reference 2 as well. We changed the description in our article to “2-4 replicates”, since unstimulated macrophages’ time point 24h has 4 sequenced replicates.

1. Can the authors provide some script or functions for users to process the raw data into MIREyA input format? For example, the first column in *data/DE_mirnas_expression.tsv*, is the miRNA names, but not in the format of mostly used.

It is unclear what exactly the reviewer means by mentioning the raw data. If the reviewer has in mind any specific format we can provide a script for the conversion. For now, one may use miRNA names in any format. The only thing to keep in mind is that they should be named consistently in all input files. Sequences of mature miRNAs may be downloaded from miRBase database: http://www.mirbase.org/ftp.shtml. Seed sequences of miRNAs could be retrieved from, e.g. http://www.targetscan.org/cgi-bin/targetscan/data_download.cgi?db=mmu_72.

1. The example expressions are normalized, it would be clear to mention when explaining *-ge* on page 4.

We specified that gene expression is expected to be normalized.

1. Improve the logging and exit message of running the pipeline. 

Thank you for the suggestions. We added more logging in the latest release. The pipeline now exits if in seed_match_needle mode there is no match of miRNA seeds in the genome and genome as fasta files per chromosome are provided in incorrect format.

**Optional:**

1. The current code looks like in the script format, wrapping them into package and release to pypi may improve the package popularity and availability. The code looks much like customer scripts for analysis for case use data, generation of them into wider usage. 

We believe that for now it is out of the scope of this work. This is an option to consider in the future.

1. Some genes have multiple copies across the genome, when calculating the correlation between gene expression and enhancer/miRNA, will copy/genomic coordinate be given to the correlation test? At least 3 line expression data about Ccdc59 in the example expression data data/DE_gene_expression.tsv, which one is used in the correlation test?

The expression dataset we use represents promoters not genes obtained by CAGE-seq technique. It has been shown previously that on average each human gene has 5 promoters. So in our dataset “copies” represent different promoters of genes. The pipeline will consider them separately. If one has a similar dataset, they may choose a maximum correlation coefficient per gene after running the pipeline. Alternatively, it is possible to sum promoter expression into gene expression from the beginning and pass it to the pipeline using a flag -ge. In the example dataset we used promoter separetely.

1. I could not reproduce the first example 1 on page 5:

- git clone git@github.com:veania/MIREyA.git
- Rename the out folder within this repo code, then create another out for testing,
- python src/run_mireya.py -d seed_match_needle -e data/enhancers.macrophages.Mtb.mm9.fasta -o out/seed_match_needle_out/ -ge data/DE_gene_expression.tsv -me data/DE_mirnas_expression.tsv -ei data/enh.gene.assoc.sign.tsv -m data/DE_mirna_mature_seqs.fa -g data/db -s data/seeds_seq_forward_short -sr data/seeds_seq_reverse_compl_short -eb data/enhancers.macrophages.Mtb.bed -ms data/mature_seqs/

  

 First error in step 1:

- Error: Unable to open file out/seed_match_needle_out//Mir146_seed_pos_in_genome. Exiting.

Even with this error, the pipeline keeps running without exit.  For the step 2, I got another error:

- File 'Mir146_enh_with_seeds.bed' has size 0. Returning a NULL data.table.
- Execution halted
- Calculation is finished. Please find the results in mir_enh_gene_trios.tsv

We repeated your actions on two computers and could not catch this error. To try to reproduce this error we need information about versions of your Linux distribution, Python, bedtools. Nevertheless, we did add a message about exiting for the case if this error occurs.

# Peer review: Reviewer response for version 1

**DOI:** 10.5256/f1000research.31129.r82451

**Published:** 2021-04-19

**Authors:** Michiel Jan Laurens De Hoon

**Affiliations:** 1 RIKEN Center for Integrative Medical Sciences, Yokohama, Japan

**Summary:**

 This manuscript describes a novel software tool to detect interactions between miRNAs, enhancers, and the genes they regulate. Though the analysis performed by this software pipeline seems straightforward, it is a welcome addition as a bioinformatics tool, as the activation of enhancers by miRNAs has not been studied in much detail compared to the well-known mechanism of gene regulation by miRNAs via the RNAi pathway.

**Comments and suggestions for improvement:**

- Some of the references to the scientific literature are appropriate but quite old (for example, references 1, 9, and 26). Can these references be updated?
- On page 4, I assume that "percent identity (PI) > 0.5" should be "percent identity (PI) > 50".
- On page 5, the format and contents of the output file mir_enh_gene_trios.tsv is not clearly explained.
- In Table 2, should a column with the number of enhancers be added (same as in Table 1)?

**Optional:**

- In the results, what is the evidence that miRNA-gene interactions shown in Table 1 and 2 are true? If no such evidence is available, can the pipeline be used to rediscover the miRNA/enhancer/gene interactions described in reference 3, 4, and 5? I understand that this may be difficult as currently there are not many examples available together with suitable expression data sets, but perhaps it can be shown that at least the first step in the pipeline (detecting miRNA-enhancer interactions by Needle, MiRanda, or Triplexator) gives results consistent with what is described in these three references? That would make the paper more convincing.
- As a general point: Are both Python and R needed to run the pipeline? Could the pipeline be designed such that only Python or only R are needed?

Are the conclusions about the tool and its performance adequately supported by the findings presented in the article?

Partly

Is the rationale for developing the new software tool clearly explained?

Yes

Is the description of the software tool technically sound?

Yes

Are sufficient details of the code, methods and analysis (if applicable) provided to allow replication of the software development and its use by others?

Yes

Is sufficient information provided to allow interpretation of the expected output datasets and any results generated using the tool?

Partly

Reviewer Expertise:

Computational genomics, bioinformatics, microRNA regulation.

I confirm that I have read this submission and believe that I have an appropriate level of expertise to confirm that it is of an acceptable scientific standard, however I have significant reservations, as outlined above.

# Response

**Published:** 2021-08-02

**Authors:** Anna Elizarova

**Affiliations:** Research Center of Biotechnology, Institute of Bioengineering, Russian Academy of Sciences, Russian Federation

Thank you for leaving the review.

 As for the questions:

- Some of the references to the scientific literature are appropriate but quite old (for example, references 1, 9, and 26). Can these references be updated?

We replaced reference 1 with a reference of a more recent review. The 9th reference is a reference to the original article of miRanda tool which is rather old but still functioning and suitable for our needs. The 26th reference is the most recent (2014) article to cite for miRBase database.

- On page 4, I assume that "percent identity (PI) > 0.5" should be "percent identity (PI) > 50".

We replaced 0.5 with 50 in the text of the article and we replaced PIs in the algorithm with values from 0 to 100 instead of 0 to 1.

- On page 5, the format and contents of the output file mir_enh_gene_trios.tsv is not clearly explained.

We added the following explanation to the main text of the article:

 One line in the output file corresponds to one trio of a mature miRNA, an enhancer and a gene (“mature_mirna”, “Gene.Name”, “enhancer” columns). Columns “corr(miRNA, gene)” and “p.value adj” correspond to Spearman’s correlation coefficient of the miRNA and the gene expression and FDR respectively. “PI” column corresponds to percent identity and is present only in the output of seed_match_needle approach.

- In Table 2, should a column with the number of enhancers be added (same as in Table 1)?

We added a column with the number of enhancers to table 2.

**Optional:**

- In the results, what is the evidence that miRNA-gene interactions shown in Table 1 and 2 are true? If no such evidence is available, can the pipeline be used to rediscover the miRNA/enhancer/gene interactions described in reference 3, 4, and 5? I understand that this may be difficult as currently there are not many examples available together with suitable expression data sets, but perhaps it can be shown that at least the first step in the pipeline (detecting miRNA-enhancer interactions by Needle, MiRanda, or Triplexator) gives results consistent with what is described in these three references? That would make the paper more convincing.

As for the evidence that miRNA-gene interactions shown in Table 1 and 2, we do understand that the output of our pipeline needs to be validated experimentally. The redescovering  of  the miRNA/enhancer/gene interactions described in reference 3, 4, and 5 is impossible since there’s no expression dataset available in these references. MiRNA expression is highly dependent on cell type and conditions, thus any other expression dataset would have a different set of expressed miRNAs which might not include miRNAs studied in the papers. Repeating the first step (miRNA/enhancer interaction prediction) in the pipeline is impossible for the references 4, 5, since they describe other mechanisms of gene activation by miRNA without enhancer activation. The miRNA MiR-24-1 is apparently present in the sequence of the enhancer in the reference 3, because the enhancer contains a gene sequence of the miRNA as described in the article. Therefore an alignment of miRNA to the enhancer automatically will be successful.

- As a general point: Are both Python and R needed to run the pipeline? Could the pipeline be designed such that only Python or only R are needed?

We started developing our pipeline as a set of R and bash scripts and added Python later due to the need of Biopython functions to work with fasta files. We will consider using only Python for MIREyA as a further step.
